# Long-term secular trends in dermatomyositis and polymyositis mortality in the USA from 1981 to 2020 according to underlying and multiple cause of death mortality data

**DOI:** 10.1186/s13075-022-02963-y

**Published:** 2023-01-31

**Authors:** Pengyan Qiao, Qianyu Guo, Jinfang Gao, Dan Ma, Sumiao Liu, Xiang Gao, Tsung-Hsueh Lu, Liyun Zhang

**Affiliations:** 1 Department of Rheumatology, Third Hospital of Shanxi Medical University, Shanxi Bethune Hospital, Shanxi Academy of Medical Sciences, Tongji Shanxi Hospital, Taiyuan, 030032 Shanxi China; 2 Department of Public Health, National Cheng Kung University, Tainan, Taiwan

**Keywords:** Dermatomyositis, Polymyositis, Autoimmune inflammatory myopathy, Mortality trends, Underlying cause of death, Multiple causes of death

## Abstract

**Background:**

People with dermatomyositis (DM) or polymyositis (PM) often die from cancer, pulmonary, cardiac complications, or infections. In such cases, DM or PM might not be designated as the underlying cause of death (UCD) for mortality tabulation. In this study, we investigated DM/PM mortality trends in the USA from 1981 to 2020 with respect to UCD and multiple causes of death (MCD) data.

**Methods:**

We used the MCD data to identify all deaths with DM or PM mentioned anywhere on the death certificate and as the UCD in the USA from 1981–1982 to 2019–2020. We calculated age-adjusted mortality rates (AAMRs) and annual percentage changes (APCs) based on joinpoint regression analysis.

**Results:**

We identified 12,249 (3985 with DM and 7097 with PM) and 23,608 (8264 with DM and 15,344 with PM) people who died between 1981 and 2020 according to the UCD and MCD data, respectively. For DM, the APC was − 6.7% (from 1981–1982 to 1985–1986), − 0.1% (from 1985–1986 to 2003–2004), and − 1.9% (from 2003–2004 to 2019–2020) according UCD and was − 1.2% (from 1981–1982 to 2003–2004), − 2.5% (from 2003–2004 to 2015–2016), and 2.8% (from 2015–2016 to 2019–2020) according MCD. For PM, the APC was 1.9% (from 1981–1982 to 1989–1990), − 2.3% (from 1989–1990 to 2005–2006), and − 5.2% (from 2005–2006 to 2019–2020) according UCD and was 1.3% (from 1981–1982 to 1991–1992) and − 4.1% (from 1991–1992 to 2019–2020) according MCD.

**Conclusion:**

We identified two times as many DM/PM deaths using the MCD as those identified using the UCD. Similar downward DM/PM mortality trends were noted according to UCD and MCD. However, the year of significant decline in PM mortality was about 10 years earlier according to MCD than those according to UCD.

## Introduction

Dermatomyositis (DM) and polymyositis (PM) are systemic inflammatory autoimmune myopathies affecting the skeletal muscles, skin, and other organs, with high morbidity and mortality [1, 2]. According to a 2012 systemic review by Marie, the mortality rates of DM and PM have declined because of earlier diagnosis and the use of immunosuppressive agents [2]. Despite the improvement in survival rates, people with DM/PM still have a mortality rate three times higher than that of the general population [1]. Several mortality studies published after 2012 have indicated the higher mortality risk among people with DM/PM persisted [3–9]. However, only two population-based studies have examined the changes in DM/PM mortality over time: one used integrative healthcare data from 1997–2005 and 2006–2014 in British Columbia (BC), Canada, and the other study used electronic medical records from general practitioners from 1999–2006 and 2007–2014 in the United Kingdom (UK) [6, 9]. Nevertheless, the number of deaths in each study was low (303 in the BC study and 114 in the UK study), which hindered further analysis by sex and age. One early US study identified 1986 DM/PM deaths from 1968 to 1978 by using mortality data compiled by the National Center for Health Statistics (NCHS) and reported increases in the annual age-adjusted mortality rates of both men and women [10]. No study has examined the long-term secular trends in the annual DM and PM mortality rates in the USA over the past 4 decades using the NCHS mortality data.

**Fig. 1:**
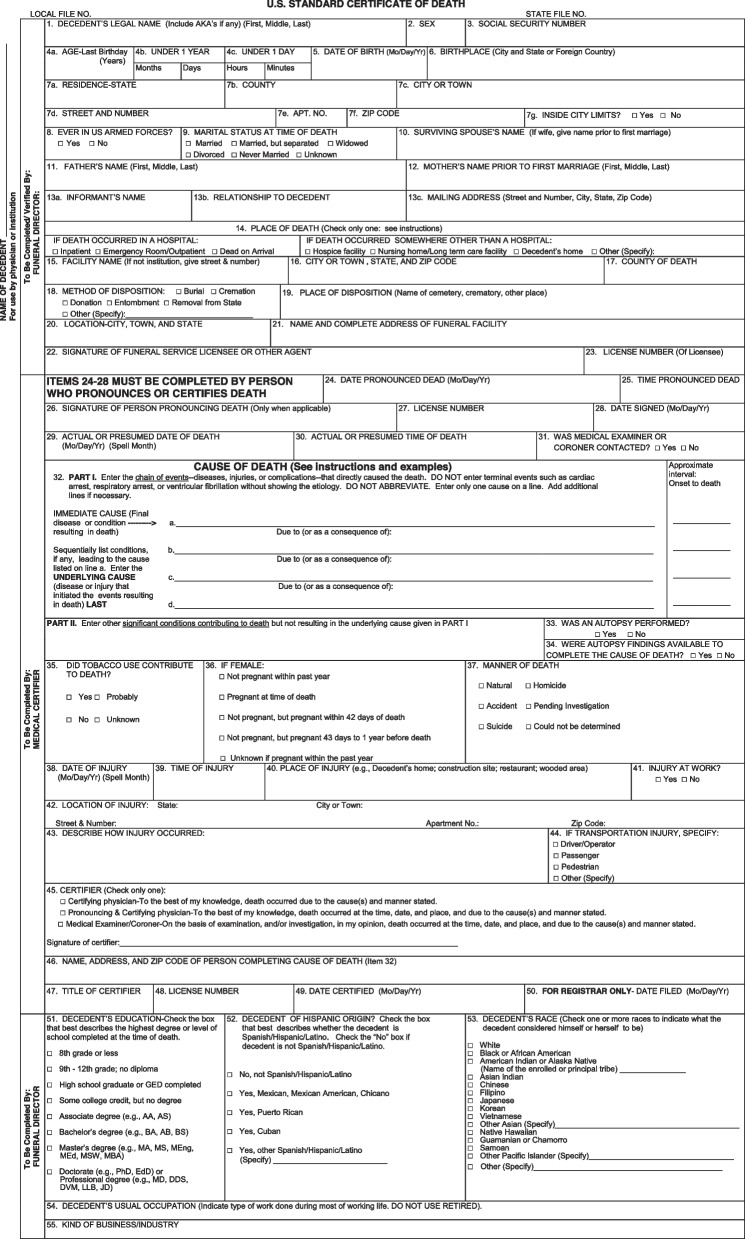
Cause of death form in the US standard death certificate

People with DM/PM often die from cancer or from pulmonary or cardiac complications or infections [1, 2]. In such cases, DM and PM might not be designated as the underlying cause of death (UCD) for mortality tabulation. Official published mortality data [11] are compiled according to the UCD, which is defined by the World Health Organization (WHO) as (a) the disease or injury that initiated the train of morbid events leading directly to death or (b) the circumstances of the accident or violence that produced the fatal injury [12]. To ensure the comparability of cause of death (COD) statistics across countries, the WHO designed a standard international form of the medical certificate of COD (Fig. 1) and developed coding instructions for selecting the UCD to tabulate mortality [12]. For example, in the following three cases, the DM/PM would be selected as the UCD in case 1 only. However, the DM/PM would be counted as DM/PM-related deaths if we used multiple cause of death (MCD) data compiled by the NCHS, in which all causes of death recorded on each death certificate by medical certifiers would be included [13].

### Case 1

Part I

- Sepsis
- Aspiration pneumonia
- Dysphagia
- Dermatomyositis/polymyositis

Part II Hypertension

### Case 2

Part I

- Respiratory failure
- Lung cancer
- .
- .

Part II Dermatomyositis/polymyositis

### Case 3

Part I

- Arrhythmia
- Acute myocardial infarction
- Hypertension, dermatomyositis/polymyositis
- .

Part II

According to a study analyzed DM/PM related mortality in state of Sao Paulo, Brazil, between 1985 and 2007, of 318 DM deaths and 316 PM deaths identified according to MCD data, 57% (180/318) and 54% (170/316), respectively, the DM/PM were designated as the UCD [14]. No DM/PM mortality study has used US MCD data to estimate the burden of DM/PM mortality. In this study, we investigated whether the long-term secular trends in DM/PM mortality in the USA from 1981 to 2020 according to the UCD data differed from those according to MCD data.

## Methods

### Data sources and case definition

We used the MCD files compiled by the NCHS to identify all deaths with a mention of DM and PM anywhere on the death certificate in the USA from 1981 to 2020 [13]. The NCHS MCD data account for up to 20 causes of death (CODs) recorded on each death certificate by medical certifiers. The MCD data also include assigned UCD, demographic information (sex, age, and race), and information on the place of residence and place of death of each decedent. The database captures more than 99% of deaths among US residents in all 50 states and the District of Columbia [13]. The NCHS used the International Classification of Diseases, Ninth Revision and Tenth Revision (ICD-9 and ICD-10, respectively) for COD coding from 1979 to 1998 and from 1999 to 2020, respectively. The ICD-9 codes for DM and PM are 710.3 and 710.4, respectively. The ICD-10 codes M33.0, M33.1, and M33.9 are for DM and M33.2 for PM.

### Mortality rates

To have fair comparisons across years, age-adjusted mortality rates (AAMR) deaths per 1 million people for DM and PM were calculated. Following previous mortality studies in rheumatology field, [15–18] we used population age distribution of year 2000 in the USA as standard population for adjustment [19]. Because of small number of DM/PM deaths, we used four age groups (0–44, 45–64, 65–74, and ≥ 75 years) to calculated age-adjusted mortality rates and combined 2 years together as the unit of observation (1981–1982, 1983–1984, etc.). We thus had a total of 20 observations for 40 years.

### Statistical analysis

To test if the mortality trend had an apparent change is statistically significant, the Joinpoint regression program version 4.9.1.0 (National Cancer Institute) was used to identify inflection points and annual percentage changes (APCs) using linear regression [20, 21].

## Results

We identified 8264 and 15,344 people who died between 1981 and 2020 with mention of DM and PM on their death certificates according to the MCD data, respectively; 3985 and 7097 of them the DM and PM was assigned as the UCD, respectively. The UCD and MCD overlap rate was 48% for DM and 46% for PM. The number of deaths, AAMRs, and UCD/MCD (U/M) ratio for each study year are illustrated in Table 1 (both sexes), Table 2 (males), and Table 3 (females). The overall AAMRs (deaths per 1 million people) for 1981 through 2020 combined for females was 0.42 and 0.87 according to UCD and MCD, respectively for DM and 0.69 and 1.46, respectively for PM, which was higher than those for males: 0.24 and 0.52, respectively for DM and 0.52 and 1,15, respectively for PM.

**Table 1:** Number of deaths (No) and age-adjusted mortality rate (death per 1 million people)) of dermatomyositis and polymyositis according to underlying cause of death (UCD) and multiple causes of death (MCD) in the USA from 1981–1982 to 2019–2020, both sexes

	Dermatomyositis								Polymyositis						
	UCD			MCD					UCD			MCD			
Year	No	Rate		No	Rate		U/M		No	Rate		No	Rate		U/M
1981–2020	3985	0.34		8264	0.71		0.48		7097	0.61		15,344	1.32		0.46
1981–1982	208	0.49		421	0.99		0.50		328	0.79		755	1.83		0.43
1983–1984	208	0.40		397	0.93		0.44		342	0.79		796	1.85		0.43
1985–1986	208	0.38		394	0.89		0.43		368	0.84		876	2.00		0.42
1987–1988	177	0.38		404	0.88		0.43		376	0.83		912	2.00		0.41
1989–1990	174	0.37		394	0.83		0.44		442	0.95		946	2.02		0.47
1991–1992	168	0.34		382	0.78		0.44		408	0.84		990	2.03		0.41
1993–1994	186	0.37		423	0.83		0.44		425	0.84		944	1.86		0.45
1995–1996	182	0.35		416	0.79		0.44		384	0.73		918	1.74		0.42
1997–1998	188	0.34		402	0.74		0.47		408	0.75		875	1.61		0.47
1999–2000	213	0.38		433	0.77		0.49		433	0.77		909	1.63		0.48
2001–2002	199	0.35		441	0.77		0.45		379	0.66		778	1.35		0.49
2003–2004	227	0.38		445	0.75		0.51		398	0.67		776	1.30		0.51
2005–2006	213	0.35		417	0.68		0.51		396	0.64		766	1.25		0.52
2007–2009	206	0.32		409	0.64		0.51		326	0.51		648	1.02		0.50
2009–2010	203	0.31		402	0.61		0.51		311	0.48		636	0.97		0.49
2011–2012	214	0.31		418	0.61		0.51		316	0.47		647	0.95		0.49
2013–2014	209	0.30		398	0.56		0.53		308	0.43		597	0.83		0.52
2015–2016	198	0.27		391	0.54		0.51		268	0.36		550	0.75		0.49
2017–2018	190	0.26		404	0.54		0.48		243	0.31		473	0.61		0.52
2019–2020	214	0.28		473	0.60		0.46		238	0.29		552	0.68		0.43

Using age structure of year 2000 in the USA as standard population for adjustment

**Table 2:** Number of deaths (No) and age-adjusted mortality rate (death per 1 million people)) of dermatomyositis and polymyositis according to underlying cause of death (UCD) and multiple causes of death (MCD) in the USA from 1981–1982 to 2019–2020, males

	Dermatomyositis								Polymyositis						
	UCD			MCD					UCD			MCD			
Year	No	Rate		No	Rate		U/M		No	Rate		No	Rate		U/M
1981–2020	1262	0.24		2702	0.52		0.47		2625	0.52		5790	1.15		0.45
1981–1982	68	0.38		135	0.76		0.50		118	0.68		305	1.76		0.39
1983–1984	54	0.30		139	0.78		0.38		130	0.74		314	1.77		0.42
1985–1986	54	0.28		144	0.75		0.37		150	0.84		362	2.00		0.42
1987–1988	54	0.27		144	0.72		0.37		122	0.65		320	1.70		0.38
1989–1990	62	0.30		142	0.69		0.43		152	0.79		356	1.87		0.42
1991–1992	47	0.22		126	0.61		0.37		153	0.75		382	1.89		0.40
1993–1994	57	0.26		124	0.57		0.46		156	0.73		356	1.66		0.44
1995–1996	64	0.29		138	0.62		0.46		147	0.67		330	1.51		0.44
1997–1998	57	0.25		134	0.57		0.43		148	0.63		336	1.47		0.43
1999–2000	62	0.26		121	0.49		0.52		159	0.69		336	1.43		0.48
2001–2002	69	0.27		150	0.59		0.46		134	0.55		284	1.16		0.47
2003–2004	65	0.25		127	0.49		0.51		144	0.58		267	1.07		0.54
2005–2006	61	0.22		110	0.40		0.56		131	0.49		255	0.97		0.51
2007–2009	69	0.24		140	0.49		0.48		123	0.44		226	0.81		0.54
2009–2010	67	0.22		134	0.45		0.50		117	0.41		242	0.84		0.48
2011–2012	82	0.26		142	0.45		0.58		135	0.44		263	0.87		0.51
2013–2014	60	0.18		134	0.41		0.44		125	0.40		239	0.76		0.53
2015–2016	57	0.16		119	0.34		0.47		101	0.30		217	0.65		0.47
2017–2018	68	0.20		125	0.37		0.55		98	0.27		190	0.53		0.51
2019–2020	85	0.24		174	0.47		0.50		82	0.22		210	0.57		0.39

Using age structure of year 2000 in the USA as standard population for adjustment

**Table 3:** Number of deaths (No) and age-adjusted mortality rate (death per 1 million people)) of dermatomyositis and polymyositis according to underlying cause of death (UCD) and multiple causes of death (MCD) in the USA from 1981–1982 to 2019–2020, females

	Dermatomyositis								Polymyositis						
	UCD			MCD					UCD			MCD			
Year	No	Rate		No	Rate		U/M		No	Rate		No	Rate		U/M
1981–2020	2654	0.42		5562	0.87		0.48		4472	0.69		9554	1.46		0.47
1981–1982	140	0.59		286	1.20		0.50		210	0.88		450	1.91		0.46
1983–1984	122	0.50		258	1.06		0.47		212	0.86		482	1.97		0.44
1985–1986	117	0.46		250	1.00		0.46		218	0.87		514	2.04		0.43
1987–1988	123	0.47		260	1.00		0.47		254	0.97		592	2.26		0.43
1989–1990	112	0.43		252	0.96		0.45		290	1.08		590	2.19		0.50
1991–1992	121	0.43		256	0.93		0.46		255	0.92		608	2.17		0.42
1993–1994	129	0.46		299	1.04		0.44		269	0.94		588	2.02		0.46
1995–1996	118	0.40		278	0.93		0.42		237	0.79		588	1.95		0.41
1997–1998	131	0.44		268	0.89		0.49		260	0.85		539	1.74		0.49
1999–2000	151	0.48		312	1.00		0.48		274	0.87		573	1.80		0.48
2001–2002	130	0.41		291	0.91		0.45		245	0.75		494	1.51		0.50
2003–2004	162	0.50		318	0.97		0.51		254	0.76		509	1.52		0.50
2005–2006	152	0.45		307	0.91		0.50		265	0.78		511	1.49		0.52
2007–2009	137	0.40		269	0.77		0.52		203	0.58		422	1.19		0.48
2009–2010	136	0.38		268	0.75		0.51		194	0.53		394	1.08		0.50
2011–2012	132	0.36		276	0.74		0.48		181	0.48		384	1.01		0.47
2013–2014	149	0.40		264	0.69		0.57		183	0.46		358	0.90		0.51
2015–2016	141	0.37		272	0.70		0.52		167	0.42		333	0.83		0.50
2017–2018	122	0.31		279	0.70		0.45		145	0.34		283	0.66		0.52
2019–2020	129	0.31		299	0.71		0.44		156	0.36		342	0.77		0.46

Using age structure of year 2000 in the USA as standard population for adjustment

**Fig. 2:**
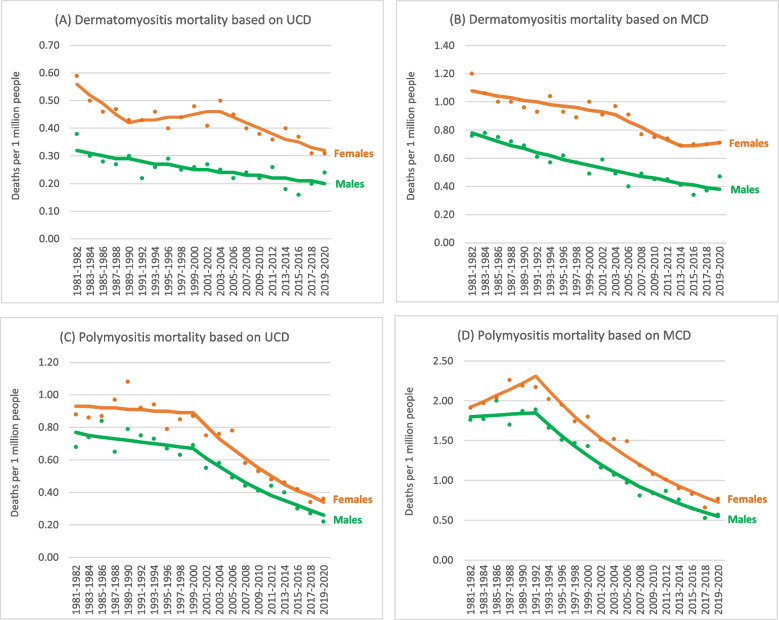
Age-adjusted dermatomyositis and polymyositis mortality rates (deaths per 1 million people) in the USA by sex according to underlying cause of death (UCD) and multiple causes of death (MCD) data from 1981–1982 to 2019–2020

The APCs and years of mortality trends by sex according to UCD and MCD are illustrated in Table 4 and Fig. 2. For DM among males, we noted one linear trend according to both UCD and MCD and the APC in AAMRs was − 1.2% and − 1.6%, respectively from 1981–1982 to 2019–2020. For DM among females, two joinpoints with three trends were identified according to both UCD and MCD. The APC was − 3.6% (from 1981–1982 to 1989–1990), 0.7% (from 1989–1990 to 2003–2004), and − 2.4% (from 2003–2004 to 2019–2020) according to UCD. The APC was − 0.7% (from 1981–1982 to 2003–2004), − 2.8% (from 2003–2004 to 2013–2014), and 0.4% (from 2013–2014 to 2019–2020) according to MCD.

**Table 4:** Annual percent change (APC) of dermatomyositis and polymyositis mortality rates based on joinpoint regression analysis according to underlying cause of death (UCD) and multiple causes of death (MCD) in the USA by sex from 1981–1982 to 2019–2020

	Trend 1				Trend 2				Trend 3		
	Year	APC	*p* value		Year	APC	*p* value		Year	APC	*p* value
Dermatomyositis, both sexes											
UCD	1981–1982 to 1985–1986	− 6.7%	0.046		1985–1986 to 2003–2004	− 0.1%	0.681		2003–2004 to 2019–2020	− 0.1%	< 0.001
MCD	1981–1982 to 2003–2004	− 1.2%	< 0.001		2003–2004 to 2015–2016	− 2.5%	< 0.001		2015–2016 to 2019–2020	2.8%	0.256
Dermatomyositis, males											
UCD	1981–1982 to 2019–2020	− 1.2%	< 0.001								
MCD	1981–1982 to 2019–2020	− 1.9%	< 0.001								
Dermatomyositis, females											
UCD	1981–1982 to 1989–1990	− 3.6%	0.037		1989–1990 to 2003–2004	0.7%	0.448		2003–2004 to 2019–2020	− 2.4%	0.001
MCD	1981–1982 to 2003–2004	− 0.7%	0.021		2003–2004 to 2013–2014	− 2.8%	0.048		2013–2014 to 2019–2020	0.4%	0.842
Polymyositis, both sexes											
UCD	1981–1982 to 1989–1990	1.9%	0.105		1989–1990 to 2005–2006	− 2.3%	< 0.001		2005–2006 to 2019–2020	− 5.2%	< 0.001
MCD	1981–1982 to 1991–1992	1.3%	0.115		1991–1992 to 2019–2020	− 4.1%	< 0.001				
Polymyositis, males											
UCD	1981–1982 to 1999–2000	− 0.7%	0.262		1999–2000 to 2019–2020	− 4.6%	< 0.001				
MCD	1981–1982 to 1991–1992	0.3%	0.779		1991–1992 to 2019–2020	− 4.3%	< 0.001				
Polymyositis, females											
UCD	1981–1982 to 1999–2000	− 0.3%	0.622		1999–2000 to 2019–2020	− 4.7%	< 0.001				
MCD	1981–1982 to 1991–1992	1.9%	0.083		1991–1992 to 2019–2020	− 4.0%	< 0.001

For PM, one joinpoint with two trends were noted for both males and females according to UCD and MCD. For males, the APC was − 0.7% (from 1981–1982 to 1999–2000) and − 4.6% (from 1999–2000 to 2019–2020) according to UCD and was 0.3% (from 1981–1982 to 1991–1992) and − 4.3% (from 1991–1992 to 2019–2020) according to MCD. For females, the APC was − 0.3% (from 1981–1982 to 1999–2000) and − 4.7% (from 1999–2000 to 2019–2020) according to UCD and was 1.9% (from 1981–1982 to 1991–1992) and − 4.0% (from 1991–1992 to 2019–2020) according to MCD.

## Discussion

### Main findings

In this study, we identified two times as many DM/PM deaths using the MCD data as identified using the UCD data. Persistent downward DM mortality trends for both sexes over the past 4 decades were noted and the patterns were similar according to UCD and MCD. With regard to PM mortality trends for both sexes, the year of significant decline were in late 1990s according to UCD; nevertheless, were in early 1990s according to MCD. The magnitude of decline in PM mortality was larger than that in DM mortality.

### Interpreting the findings in the context of previous studies

The main results of two previous population-based cohort studies of DM and PM mortality are summarized in Table 5 [6, 9]. We calculated the mortality rate ratios between early and late observation periods in the two studies and found that the extent of decrease in DM mortality was greater than that in PM mortality in the UK study; no obvious differences were noted in the BC study. However, in the present study, the extent of decrease in PM mortality was more prominent than that in DM mortality.

**Table 5:** Numbers of deaths (No), mortality rates (deaths per 1000 person-years), and rate ratios (RRs) in two periods according to two population-based cohort studies

Authors, year		(1) Period 1			(2) Period 2			(2)/(1)	
(Location)	Myopathy	No	Rate		No	Rate		RR	(95% CI)
Li et al., 2020^a ^[6]	Dermatomyositis	70	80.1		73	51.3		0.64	(0.46–0.89)
(BC, Canada)	Polymyositis	79	58.6		81	35.4		0.60	(0.44–0.82)
D’Silva et al., 2021^b ^[9]	Dermatomyositis	33	71.5		33	49.1		0.69	(0.42–1.11)
(United Kingdom)	Polymyositis	23	42.1		25	34.4		0.82	(0.46–1.42)

^a^Periods 1 and 2 were 1997–2005 and 2006–2014, respectively

^b^Periods 1 and 2 were 1999–2006 and 2007–2014, respectively

Several caveats should be noted in interpreting the differences between the results of the present study and those of the two cohort studies. First, the mortality rates calculated in the cohort studies were actually case fatality rates; that is, the denominators were the numbers of patients with DM/PM diagnoses. However, the denominator of mortality rate in the present study was that of the general population, the mortality rate was affected by two components: the incidence (prevalence) rate and the case fatality rate. According to a population-based study (Rochester Epidemiology Project) in Olmsted County, the incidence of DM was 1.2 (per 100,000 person-years) in 1995–2007 and 1.1 in 2008–2019, no evidence of a change over time [22]. Therefore, the decline in DM/PM mortality rates observed in this study was mainly due to the reduction in case fatality rate. As indicated by Li et al., the early use of disease-modifying antirheumatic drugs (rituximab, methotrexate, azathioprine, and mycophenolate mofetil) and the increasing use of intravenous immunoglobulin might be key factors affecting the decline in DM/PM mortality in recent decades [6].

The findings of this study further indicated that the extent of decline in mortality rates was larger in PM (APC for males was − 4.6% and − 4.3% according to UCD and MCD, respectively) than those in DM (APC for males was − 1.2% and − 1.9%, respectively). However, no such difference was noted in UK and BC study (Table 5) [6, 9]. One plausible explanation was the differences in the robustness of diagnosis of DM/PM between those based on the death certificate versus those based on hospital records (will be discussed later in the limitation section).

The second caveat was that the years of observation differed between the two cohort studies with this study. The time span was 1996 through 2014 in two cohort studies and was 1981 through 2020 in the present study, which hindered valid comparisons. The trends in DM and PM mortality rates might change across study periods. An early study conducted in the USA reported that the age-adjusted DM/PM mortality rates increased from 1968 to 1978, [10] which is in contrast with the decreasing mortality trends since 1981 revealed in the present study.

With regard to the interpretation of the mortality rates estimated according to UCD and MCD data, it is better to examine the instruction depicted in the US standard death certificate: “Enter the chain of events – diseases, injuries, or complications – that directly cause death in Part I” and “Enter other significant conditions contributing to death but not resulting in the underlying cause given in Part I” (Fig. 1). If DM/PM were recorded by certifying physicians in Part II, DM/PM were less likely been designated as the UCD. The proportion of DM/PM as the UCD among those with mention of DM/PM could be a proxy measure of case fatality rate of DM/PM.

In this study, the proportion of DM/PM as UCD among MCD was 48% for DM and 46% for PM and about the same through the study period. The proportion was lower than that in Brazil, which was 57% for DM and 54% for PM [15]. The first probable explanation for the difference was that people with DM/PM in the USA were better treated than their counterparts in Brazil and therefore had lower DM/PM case fatality rates. The second possible explanation was that the case fatality rates of DM/PM were similar in two countries; nevertheless, the US physicians were more likely than their counterpart Brazil physicians to record DM/PM in part II of the death certificate. Study has indicated that physicians in different countries had different habits in recording diabetes in the part II of the death certificate [23].

### Strengths and weaknesses

One strength of the present study is its use of nationwide population-based mortality data collected across 40 years to examine long-term trends in DM and PM mortality. This is also the first study to compare the DM and PM mortality trends between those according to UCD versus those according to MCD.

However, this study has several limitations that should be noted. First, some physicians might underreport DM/PM on death certificates. There was no study specifically examined the magnitude of underreporting of DM/PM on the death certificate. According to two studies assessed the underreporting of systemic lupus erythematosus (SLE) on the death certificate, only 40% of people with SLE died, the physicians recorded SLE on the death certificate. The underreporting was higher as the age increased and among people with cancer [24, 25]. However, because the main aim of this study was to examine mortality trends, the underreporting rate is unlikely to have systematically biased the results over time if there were no specific interventions on the certification behaviors.

Second, the validity of using ICD codes to identify DM/PM should be concerned. As it is common that other systemic inflammatory diseases and inherited muscle diseases including muscular dystrophies and metabolic myopathies could be misdiagnosed as idiopathic inflammatory myopathies (IIM). Therefore, it is possible that a decedent assigned with an ICD code of DM/PM as the underlying cause of death or indirect cause of death might actually have no DM/PM. According to a validity study of using ICD code to identify DM, the sensitivity and positive predictive value (PPV) for multiple ICD-9 codes 710.3 in the outpatient setting were 0.89 and 0.35, respectively. The PPV for primary and secondary inpatient codes of 710.3 was 0.95 and as high as 0.80 [26]. An UK study assessed the validity of using ICD-10 codes to identify IIM in hospital episode statistics data indicated sensitivity of 0.73 and PPV of 0.73 [27]. That is to say that the validity of ICD codes in inpatient data to identify people with IMM is acceptable and most of the people died with DM/PM diagnosis recorded on the death certificates were issued by the physicians in hospital. The over-diagnosis of DM/PM on the death certificate might not be high.

Third, as the population in the USA is aging, the proportion elderly decedents with DM/PM increased across the four decades. Study has indicated that the use of age distribution in 2000 in the USA (a relatively younger age structure than in 2020) as standard for calculation of AAMRs would result in a lower estimation of the real mortality rates [28].

## Conclusion

Using the MCD data, we identified two times as many DM/PM deaths as we could identify using the UCD data. The pattern of downward DM/PM mortality trends were similar between those according to UCD with those according to MCD. However, the year of significant decline in PM mortality was about 10 years earlier according to MCD than those according to UCD. The extent of decline in PM mortality was more prominent than that in DM mortality.

## Data Availability

The data are available upon request to the corresponding author.

## Abbreviations

AAMR: Age-adjusted mortality rate
APC: Annual percent change
DM: Dermatomyositis
*ICD-9*: International Classification of Diseases Ninth Revision
*ICD-10*: International Classification of Diseases Tenth Revision
IIM: Idiopathic inflammatory myopathies
MCD: Multiple causes of death
NCHS: National Center for Health Statistics
PM: Polymyositis
RR: Rate ratio
SLE: Systemic lupus erythematosus
UCD: Underlying cause of death
95% CI: 95% Confidence interval
